# The moderating role of environment-related technologies in the link between transportation infrastructure, agricultural, and environmental contamination in highly congested countries of the world

**DOI:** 10.1080/21645698.2025.2499998

**Published:** 2025-05-04

**Authors:** Wanzhen Xu, Arshad Ali

**Affiliations:** a Institute of Environment & Development (LESTARI), University Kebangsaan Malaysia, Selangor, Malaysia; b School of Economics and Management, Northeast Agricultural University, Harbin, China

**Keywords:** Agriculture sector, CS-ARDL, environmental emission, environmentally relevant technologies, transportation

## Abstract

Densely populated countries have greater demand for agricultural output, greater vehicle usage, and busier traffic, so these countries devote more agricultural land to agricultural production and invest more in transportation infrastructure, resulting in higher environmental emissions. Most studies in literature assume that environment-related technologies directly diminish environmental contamination, while this study suggests that environment-related technologies indirectly mitigate environmental hazards through interactions with agricultural land use, agricultural value addition, and transportation infrastructure. Thus, this study determines to disclose the influence of agricultural land use, agricultural value added, and transportation infrastructure investment on ecological damage in countries with the highest population density during the period 1990–2022; and the sole moderating effect of environment-related technologies in the association of the proposed regressors with CO_2_ emissions. Results illustrate that agricultural land use and investment in transportation infrastructure contribute significantly to CO_2_ emissions, but only in the long term. However, agriculture value addition strongly promotes environmental contamination in both the short and long term. Furthermore, in the long run, the reversed U-designed EKC premise holds only for the top five most populous countries. Environmental contamination can be mitigated through environmentally relevant technologies when interacting with agriculture land use, value addition of agricultural and investment in transportation infrastructure. Finally, (>-1.115) is the estimated threshold level of environment-related technologies, at which agricultural land use, agricultural value added, and transportation infrastructure can significantly improve environmental quality. Policymakers in densely populated countries should prioritize the adoption of environmentally friendly technologies in agriculture and transportation to achieve environmental sustainability.

## 1. Introduction

Fossil fuels such as natural gas, oil and coal have been the world’s primary energy source for 200 years, powering economies and meeting 80% of the world’s energy demand. The economic prosperity of emerging economies has been strongly dependent on fossil fuel energy used in the manufacturing, transportation and agricultural sectors for more than 50 years. However, nonrenewable energy sources such as fossil fuels emit large amounts of carbon dioxide, severely exacerbating climate change and causing millions of untimely expiries by polluting native air.^1^ Pipelines, rail, shipping, aviation and roads are transport activities associated with global transport infrastructure, powered by the combustion of fossil fuels, which emit 16.2% of greenhouse gases.^2^ In many of the world’s countries with the highest population densities, the transport sector has the highest emissions, with transport infrastructure responsible for 21% of global environmental emissions.^3^ The transport sector in the most densely populated countries is the main cause of environmental emissions, accounting for 21% of global CO_2_ emissions. Despite the achievement of existing and assured strategies, carbon emissions from transport are expected to continue to grow by nearly 20% by 2050. The completely zero emissions target pursued by the Strive policy is unlikely to be attained and can only be succeeded by 70% at best. This is because as population density increases and the economy develops, people’s demand for goods, desire to travel and ways of travel are also expanding. Global traffic is projected to more than double by 2050 from 2015 levels, amid a dizzying array of new challenges.^3^ Growing travel demand will outstrip technological progress in decarbonizing transportation. It is predicted that the decarbonization targets of the Paris Agreement will not be attained by 2050 unless aspirations are scaled down to more sustainable levels. The main cause of carbon emissions is the use of fossil fuels, and currently 95% of the transportation sector relies on fossil fuels. With the resumption of passenger and freight activities after the COVID-19 pandemic, CO_2_ emissions from the transport sector boosted by 3% in 2022 compared to the previous (COVID-19) year.^4^ Countries with the highest population density have higher transport demands, which leads to higher environmental emissions. As World population and incomes continue to grow over the next decade, World transportation demand will rise as more and more individuals can afford cars, trains and planes.^5^ By 2070, world passenger traffic is projected to two-fold, car possession is estimated to rise to 60%, and demand for air passengers and cargo will tripartite.^4^ The boom in green cars and environment-related technological innovations could reduce emissions from passenger vehicles, and therefore, the world is currently shifting toward these low-carbon technologies.^6,7^ The burning of fossil fuels in the transportation sector – planes, trains, ships, trucks and cars – is the leading cause of greenhouse gas emissions.^8^ Over 94% of diesel and gasoline are petroleum-based transportation fuels that directly emit CO_2_. It is expected that by 2040, densely populated countries such as Indonesia, China, the United States and India will have to gradually phase out conventional cars. The 2050 Net Zero Emissions (NZE) scenario calls for a reduction in annual transport CO_2_ emissions of more than 3% by 2030. The goal of reducing emissions and developing low- and zero-emission vehicles can be achieved through the use of environmentally relevant technologies. As the United Nations^9^ recently reported, driving the global transition to sustainable transport requires immediate transformative action. Improving energy efficiency by adopting the latest environment-related technologies can mitigate emission levels from electric vehicles and buses, thereby achieving zero-carbon energy production in the transportation sector. Adopting environmentally friendly technologies to reduce CO_2_ emissions from transport can be a strategic step toward achieving the Sustainable Development Goals and the Paris Agreement. Furthermore, environmental technologies can facilitate the development of more sustainable and cleaner substitutes to conventional fossil fuel transport, thereby improving energy efficiency and dropping fossil fuel transport emissions.^10^

Global warming is caused by human activities and is likely to be exacerbated through two important sectors: transportation and agriculture. 12% of global greenhouse gas emissions and 21% of carbon dioxide emissions come from the agricultural sector and the transport sector, respectively. However, the agricultural sector produces large amounts of potent greenhouse gases such as nitrous oxide (N_2_O) and methane (CH₄), which have a stronger warming effect than carbon dioxide.^11^ A quarter of greenhouse gas emissions come from agriculture, land use and forestry, which pollute the environment and contribute to climate change. The main sources of agricultural emissions are land-use conversions, such as methane from livestock and rice production, nitrous oxide from the use of synthetic fertilizers, and deforestation on farms.^12^ It is noteworthy that the countries with the highest population density are also the ones with the highest greenhouse gas emissions from the agricultural sector. India is the world’s second most populous country, and its agricultural production emits 700 million tons of carbon dioxide equivalent each year, making it the largest agricultural producer of carbon dioxide emissions. The largest sources of environmental emissions from Indian agriculture are rice cultivation and enteric fermentation.^13^ The three most populous countries, China, India and the United States, have high agricultural greenhouse gas emissions, accounting for 42.6% of total emissions. Likewise, the five most populous countries – Indonesia, China, Brazil, the United States and India – produce 51% of global greenhouse gas emissions from the agricultural sector. The primary causes for the expansion in greenhouse gas emissions in these countries are the promotion in population density and dietary tendencies.^14^ More likely, as the global population grows and diets diversifies, nutritional needs will increase. World productivity levels are under stress due to severe depletion of natural resources such as soil, water and biodiversity, deteriorating ocean conditions and stagnant crop yields. In 2020, there were about 690 million hungry people, equivalent to 8.9% of the world’s population, with 60 million new people added in the past five years. By 2050, food security challenges may become even more severe, with 70% of the food needs of about 9 billion people worldwide determined by food supply.^15^ Indeed, the transition from traditional extensive agri-food systems to advanced powerful production systems has guaranteed an upsurge in agricultural productivity, and this transition is described by an expansion in on-farm energy use.^16^ Agricultural machinery such as irrigation pumps, harvesters, tractors and non-agricultural machinery such as fishing vessels and forestry machinery can utilize fossil fuels as direct energy inputs to the agricultural sector.^17^ A key factor in agricultural production and growth is the use of fossil fuel energy on farms. Food distribution, crop management, fertilizer production and machinery production are all aspects of agriculture based on fossil fuel energy use and account for 5% of global greenhouse gas emissions.^18^ Agriculture’s reliance on fossil fuels can be reduced by increasing energy efficiency through the adoption of environment-related technologies using clean energy sources such as biofuels, wind and solar energy in the agricultural sector. Adoption of environment-related technologies can not only pave the way for building a green environment but also boost agricultural productivity levels. Farmers adopting environmentally friendly technologies based on clean energy, such as biofuel production, wind turbines and solar panels, can mitigate their reliance on costly traditional energy springs. This in turn makes the agricultural process more environmentally friendly, economically viable, and reduces operating expenses.

This study incorporates environment-related technologies, transport infrastructure, agriculture land use, added value of agricultural, and CO_2_ emissions into a framework with a particular emphasis on the five countries with the highest population density, supporting the establishment of a broad and robust pioneering framework and enriching mainstream literature. The study picked countries with the highest population density because as population growth accelerates, demand for agricultural land and products, as well as transportation vehicles and infrastructure promote, leading to higher environmental emissions. Dai et al.^19^ studied the impact of transportation infrastructure and economic growth on carbon dioxide emissions in the context of the United States. Liu et al.^20^ empirically unveiled the influence of road transportation infrastructure, railway transportation infrastructure and aviation infrastructure on environmental emissions and economic prosperity in China, Russia, India and Japan. Awad et al.^21^ carefully examined the effectiveness of agriculture, institutional quality, trade, and renewable energy use in mitigating environmental pollution in sub-Saharan African countries. Similarly, Sethi, Behera, and Sethi^22^ analyzed the role of agricultural value addition, green technology innovation, renewable energy utilization, green finance, and institutional quality in addressing CO_2_ emissions in 25 developing economies. After in-depth research, it is found that there is no study in literature that focuses on agriculture, transportation, technological innovation and environmental emissions within a single framework. However, as mentioned above, a few studies do have one or two dynamics that are consistent with this study, but none of them have the perfect framework of variables presented in this study. Another important contribution of this study is to reveal that environment-related technologies have a linear impact on environmental pollution but also affect environmental pollution in a nonlinear way through interactions with transportation infrastructure, agricultural value added, and agricultural land use, which may be uncertain but needs ideal empirical revelation. To develop a more precise and robust policy framework, this study closely examines the threshold levels of environment-related technologies that reduce or enhance environmental quality by affecting key explanatory factors, agriculture, and transportation. Finally, this study can adopt the second-generation cross-sectional autoregressive distributed lag (CS-ARDL) method, ignoring the first-generation test, which does not consider the cross-sectional correlation and slope heterogeneity of panel data, thus leading to spurious regression analysis.

## 2. Literature Review

There is a growing body of literature on the serious problems of environmental contamination and climate change. Researchers have identified various economic, social and environmental factors such as globalization, financial expansion, economic growth, population growth, urbanization, trade openness, energy consumption, institutional quality, and industrialization as causes of environmental emissions.^23–27^ The Environmental Kuznets Curve (EKC) hypothesis has been tested through a large number of panel data analysis studies, revealing the relationship between economic development and environmental emissions.^28–31^ However, the model of the relationship between economic growth and environmental damage was later extended to energy use^32–36^ Currently, demographic progress, institutional quality, urbanization, financial development, trade openness, globalization, industrialization and foreign direct investment are seen as important new factors in the model linking economic prosperity and environmental contamination.^23,37–39^ However, especially in the five countries with the highest population density, less attention has been paid to other aspects such as agricultural land use, agricultural value addition and transport infrastructure and environment-related technologies.

### 2.1. Transportation Infrastructure and Environmental Hazards Nexus

For 150 years, greenhouse gases in the atmosphere have trapped heat generated by human activities, causing the planet to warm.Fossil fuel consumption for human transportation activities is the main cause of greenhouse gas emissions, especially in densely populated countries.^40,41^ Strengthening transportation infrastructure can reduce travel costs, enhance population mobility, and directly and strongly affect environmental contamination.^19,42^ However, the improvement or deterioration of environmental quality depends on the transportation infrastructure determined by people’s demand for goods. In countries with high population density, people have higher demand for goods and are more willing to use vehicles and travel, which leads to more serious environmental emissions; while in countries with low population density, the result is the opposite. Using a narrative review approach, Herath Bandara and Thilakarathne^43^ found that traffic congestion and traffic emissions from industrial activities exacerbated respiratory diseases in Colombo, Sri Lanka. The Indian city of Delhi faces serious health risks due to traffic emissions; the Pakistani city of Lahore faces increased environmental emissions due to increased energy use in the transportation sector; and the Bangladeshi city of Dhaka faces serious environmental pollution due to urbanization and automobile growth. Similarly, Wang et al.^44^ examined the correlation between transportation infrastructure and environmental emissions from a regional perspective in China and found that the emission reduction effect was most obvious in the western and central regions, while the supporting role of transportation infrastructure in the eastern region for carbon emission reduction was relatively poor. Liu et al.^20^ concluded through the AMG method that in China and India, road and aviation infrastructure contribute significantly to environmental emissions, while railway transport infrastructure can improve environmental quality. Dai et al.^19^ used the dynamic least squares method (DOLS) to conclude that over the data range of 1990–2019, the use of renewable energy in transportation mitigated CO_2_ emissions from transportation, while the use of fossil fuels in transportation and road infrastructure increased CO_2_ emissions from transportation in the United States. Pradhan et al.^45^ used QQM and VECM methods and concluded that transport sector and institutional improvements made a positive contribution to economic growth and CO_2_ emissions in 79 developing countries during the period 2005–2022.

Numerous studies in the literature show that countries with lower population density have lower or zero environmental emissions from the transport sector. Kwilinski, Lyulyov, and Pimonenko^6^ pointed out that sustainable transport infrastructure in the Eurozone, including initiatives such as biofuels, hydrogen fuel cells, and electric vehicles, has mitigated environmental emissions. Another study by Pata et al.^46^ examined the correlation between disaggregated transport infrastructure (road, rail, and aviation infrastructure) and CO_2_ emissions and concluded that aviation infrastructure and rail infrastructure boost CO_2_ emissions, while road transport and energy taxes are effective in curbing environmental pollution in the 10 highest-income EU countries during the period 2008–2020. Dzator, Acheampong, and Dzator^47^ found through empirical research that road and rail transport infrastructure can significantly alleviate environmental emissions, while aviation infrastructure significantly exacerbates environmental pollution in OECD countries. Chi^48^ used the moment-quantile regression method (MMQR) to analyze panel data from 19 European countries from 1995 to 2020 and the results showed that increasing traffic taxes and investing in road infrastructure can help reduce environmental emissions.

### 2.2. Agricultural Value-Added and Environmental Damage Nexus

Both profitable and unprofitable energy are necessary to maintain a reasonable quality of life and an suitable agricultural food supply. Economic progress and development brought about by increased agricultural productivity are based on higher energy use, but on the other hand, higher energy use in agricultural production also pollutes the environment.^49^ Densely populated countries have a greater demand for agricultural products, so agricultural production requires more energy and generates more environmental emissions. Literature shows that agricultural value added reflects agricultural total factor productivity and is a key factor in determining the increase in environmental damage.^50–52^ Karimi Alavijeh et al.^53^ demonstrated in their study that agricultural value added in the 15 most populous developing countries is significant and positive across all quantiles of CO2 emissions using a panel quantile regression with a data span from 2004 to 2020. Usman et al.^54^ empirically demonstrated that agricultural value added has a significant asymptotic impact on environmental hazards in South Asian countries. Rehman et al.^55^ used a novel Method of Movements Quantile Regression (MMQR) to determine that agricultural value added contributes significantly to enhancing environmental pollution in Asian countries, (particularly China, India, Pakistan, Bangladesh and Indonesia), with data covering the period 1996 to 2014. Lao and Luo^56^ used the panel ARDL method from 1980 to 2020 to empirically conclude that the increase in agricultural value added and industrial progress can promote carbon dioxide emissions in South Asian and East Asian countries. Raihan and Tuspekova,^57^ found through empirical estimation using the DOLS procedure that agricultural value added in Brazil during the period 1990–2019 led to an increase in CO2 emissions, which in turn led to environmental degradation. Zhai et al.^58^ found that the higher the agricultural value added in China, India, Brazil and the United States, the greater the environmental costs they usually bear, leading to higher environmental emissions.

In contrast, agricultural value added has no substantial influence on environmental risk or significantly alleviated environmental degradation in countries with lower population density. Raihan^52^ used the ARDL method to estimate that increasing agricultural value added could significantly improve Vietnam’s environmental quality by mitigating long-term and short-term CO_2_ emissions. Likewise, as in the study by Mahmood et al.^59^ environmental quality in Saudi Arabia improves with the increase in agricultural value addition. Ridzuan et al.^60^ also asserted through empirical research that environmental emissions in Malaysia decreased with improvements in crops and fisheries. Mohammed et al.^61^ also documented that GHG emissions in the EU-27 decreased as the agricultural sector developed. Similarly, Phiri et al.^62^ used the ARDL approach to confirm that CO_2_ emissions in Zambia decreased with agricultural development.

### 2.3. Agriculture Land Use and Environmental Contamination Nexus

Densely populated countries such as China, the United States, India and Brazil are the world’s largest users of crop land to meet the demand for agricultural products from a larger population, resulting in higher environmental emissions. Tacconi and Muttaqin,^63^ pointed out through empirical research that agricultural land, especially forests, is the main cause of environmental degradation in Indonesia. Haider et al.^64^ empirically demonstrated that using a pooled mean group (PMG) approach, N_2_O emissions in developing countries such as China, India, Indonesia, Brazil, and Nigeria can be significantly improved by using more agricultural land over the data period 1980 to 2012. However, agricultural N_2_O emissions or N_2_O emissions in these countries can be directly mitigated by reducing or optimizing agricultural land use. Another study conducted by Rehman et al.^65^ using the NARDL approach supported this conclusion: the expansion of land under agricultural production increased CO_2_ emissions in Nepal from 1965 to 2018. Similarly, Gu, Li, and Santos^66^ also demonstrated that CO_2_ emissions are significantly positively correlated with urban and farmland use, and negatively correlated with forest area in China. Huang et al.^67^ also emphasized that increasing the conversion rate of agricultural land to usable land by more than 8% would significantly increase greenhouse gas emissions in developing countries in Asia.

### 2.4. Environmentally Friendly Technologies and Environmental Hazards Nexus

Environmental scientists and officials strongly recommend environmentally friendly technologies as one of the effective strategies to mitigate fossil fuel energy consumption by cultivating energy efficiency, thereby improving environmental quality.^68^ Governments and private companies around the world are vigorously promoting research and development to improve existing technologies or develop new clean technologies as a strategic development to reduce environmental harm. Chu^69^ empirically found that the ecological footprint of 20 OECD countries decreased during 1990–2015 as the development of environment-related technologies and the consumption of renewable energy led to environmental sustainability. Suki et al.^70^ also empirically demonstrated through the ARDL approach that environment-related technologies can also help reduce Malaysia’s ecological footprint and carbon emissions. Fatima, Yanting, and Guohua^71^ used the PMG panel ARDL to reveal that environment-related technologies contributed to reducing CO2 emissions in 36 OECD countries during 1990–2020. Chen et al.^72^ used the Driscoll Kraay standard error method to conclude that the improvement of material productivity and the use of environment-related technologies in 17 emerging countries from 1995 to 2019 can slow down environmental emissions. Kiani et al.^73^ also found through the ARDL method of panel data from 1991 to 2018 that in South Asia and Southeast Asian countries, environment-related technologies have an adverse impact on the ecological footprint only in the long term. Han et al.^74^ used the PMG-ARDL method to reveal that G-20 countries could reduce environmental pollution by promoting the use of renewable energy by strengthening environment-related technologies during the period 1995 to 2022.Wei et al.^75^ used the GMM method to find that both environment-related technologies and green energy had significant adverse effects on environmental hazards in 50 countries.

As mentioned above, the mainstream literature on the relationship between transportation infrastructure, agricultural land use, agricultural value added and environmental contamination shows that transportation infrastructure, agricultural land use and agricultural value added in countries with high population density stimulate environmental emissions, while the opposite is true in countries with low population density. Furthermore, most of the literature suggests that there is a direct negative correlation between environment-related technologies and environmental pollution. This study fills the gap in the literature by exploring the indirect moderating role of environment-related technologies in the linkages between transportation infrastructure, agricultural land use, agricultural value addition, and environmental contamination.

## 3. Methodology

Theoretical views on the connection between income and environmental damage put forward that the economic prosperity brought about by the use of fossil fuel energy in a country’s early industrialization period could enhance environmental contamination.^76^ But these environmental discharges are starting to decline when economic prosperity grasps a definite level during the subsequent industrialization phase.^77^ This phenomenon that economic growth and environmental emissions are positively correlated in the early stage of industrialization and adversely correlated in the second stage of industrialization is called the inverted U-shaped EKC hypothesis recommended by Grossman and Krueger.^78^ The anticipated sign of the income parameter in the early stage of industrialization is optimistic in the growth-environment correlation. The early stages of the EKC hypothesis were based on the idea that greater use of fossil fuels would increase economic productivity but, on the other hand, pollute the environment. However, the second stage of the EKC hypothesis is based on the notion that after a certain level of economic development, the use of fossil energy for economic productivity will decrease, thereby reducing pollution levels and turning the sign of the expected income coefficient in the growth-environment relationship to negative.^79^ The reduction in fossil fuel energy use during the second phase of industrialization will be attributed to the country’s adoption of environmentally friendly technologies to switch from fossil fuels to clean energy, thereby mitigating environmental emissions. This study extends the EKC hypothesis framework to include transportation infrastructure, agricultural land use, agricultural value added, and environment-related technologies as determinants of environmental effluence. Thus, the following extended EKC model is established.

(1) $$\ln CO_{2,i,t}=\alpha_{0}+\alpha_{1}\ln\mathrm{AGL}+\alpha_{2}\ln\mathrm{AG}V_{i,t}+\alpha_{3}\ln\mathrm{TR}I_{i,t}+\alpha_{4}\ln\mathrm{GD}P_{i,t}+\alpha_{5}\ln\mathrm{GD}P_{i,t}^{2}+\mu_{i,t}$$

A large amount of literature research shows that environment-related technologies play a powerful and direct role in alleviating environmental contamination. However, this study considers environment-related technologies as nonlinear to explore it’s indirect moderating role in the links between transportation infrastructure, agricultural land use, agricultural value addition, and environmental damage. Thus, the above model can be extended by interacting environment-related technologies with transportation infrastructure, agricultural land use, and agricultural value addition to establish the following model.

(2) $$\ln CO_{2,i,t}\;=\;\alpha_{0}\;+\;\alpha_{1}\ln\mathrm{AGL}\;+\;\alpha_{2}\ln\mathrm{AG}V_{i,t}\;+\;\alpha_{3}\ln\mathrm{TR}I_{i,t}\;+\;\alpha_{4}\ln\mathrm{GD}P_{i,t}\;+\;\alpha_{5}\ln{\left(\mathrm{ERT}*\mathrm{AGL}\right)}_{i,t}\;+\;\alpha_{6}\ln{\left(\mathrm{ERT}*\mathrm{AGV}\right)}_{i,t}\;+\;\alpha_{7}\ln{\left(\mathrm{ERT}*\mathrm{TRI}\right)}_{i,t}\;+\;\mu_{i,t}$$

where CO_2_, AGL, AGV, TRI, ERT and GDP stand for carbon dioxide emissions, agricultural land use, agricultural value added, transportation infrastructure, environment-related technologies and gross domestic product, respectively. In addition, ERT*AGL, ERT*AGV, and ERT*TRI represent the interaction between environment-related technologies and agricultural land use, the interaction between environment-related technologies and agricultural value addition, and the interaction between environment-related technologies and transportation infrastructure, respectively. ln stands for natural logarithm and μ indicates undetected error correction term. This study analyses 33 years of panel annual data from 1990 to 2022 for the five countries with the highest population density. The explanation and quantification of the model panel variables and their data retrieval sources are shown in Table 1. The panel dynamic data of the model, such as agricultural land use measured in percentage, agricultural value added in percentage of GDP, gross domestic product (GDP) in constant 2015 US dollars, and carbon dioxide emissions in metric tons, can be retrieved from the World Bank database. Data on transport infrastructure in constant 2015 US dollars and environment-related technologies as a percentage of all technologies can be collected from the OECD database.

**Table 1.** Panel dynamics definition, quantification and data retrieval sources.

Variable symbol	Variable definition	Data measurment	Data sources
GDP AGL	Gross Domestic Product Agriculture land use	Constant 2015 US$ Percentage of land use	World Bank database World Bank database
AGV	Agriculture value addition	Percentage of GDP	World Bank database
ERT	Environemnt related technolgoies	Percentage of all technologies	OECD database
TRI	Transport infrastructure investment	Constant 2015 US$	OECD database
CO_2_	Carbon dioxide	Metric tons	World Bank database

### 3.1. Procedure for Detecting Cross-Sectional Dependence

The cross-sectional dependence problem refers to the fact that countries within a panel are frequently connected through economic channels such as trade, financial markets, and globalization, which creates spillover effects and promotes the common development of the economies of the countries within the panel. The panel data for these countries suffer from cross-sectional dependence problems, leading to spurious regression analysis and biased estimates. Thus, it becomes crucial to detect cross-sectional dependence issues before estimating panel data regression analysis. Hence, this study adopts the CSD test of Pesaran, Ullah, and Yamagata,^80^ the LM test of Breusch and Pagan,^81^ the scaled LM test of Pesaran,^82^ and the bias-corrected scaled LM test of Baltagi, Feng, and Kao^83^ to detect cross-sectional dependence in panel data. These tests can measure the cross-sectional dependence problem in panel data using the following equations.

(3) $$\mathrm{CSD}=\sqrt{\frac{2T}{N\left(N-1\right)}}\left(\sum_{i=1}^{N-1}\sum_{K=i+1}^{N}\hat{\beta }\mathrm{ik}\right)∼N\left(0,1\right)I,k$$

CSD = (1,2,3, … .45, … … .N)

(4) $$M=\;\sqrt{\frac{2T}{N\left(N-1\right)}}\left(\sum_{i=1}^{N-1}\sum_{K=i+1}^{N}\hat{\beta }\mathrm{ik}\right)\;\left[\frac{\left(T-K\right){\hat{\beta }}_{\mathrm{ik}}^{2}-\left(T-K\right){\hat{\beta }}_{\mathrm{ik}}^{2}}{\mathrm{Var}\left(T-K\right){\hat{\beta }}_{\mathrm{ik}}^{2}}\right]$$

Here, Т denotes the data sample size or the data annual time span, and N represents the country panel size or the country cross section. Obviously, if the above tests detect that the panel data has cross-sectional correlation problems, then the traditional first-generation regression estimation methods cannot perform robust analysis. In this case, the most appropriate procedure for regression analysis is the second-generation test, which provides robust results.

### 3.2. Estimation Methods of Panel Unit Roots

The panel unit root procedure of Im, Pesaran and Shin, the panel unit root technique of Phillip and Perron, the panel unit root test of Augmented Dickey Fuller, the unit root test of Lin and Chu, the unit root process of Hadri, the Levin unit root test and the Breitung unit root method all ignore the cross-sectional dependence problem. Thus, these tests cannot provide accurate information about the level of dynamic plateau and are known as traditional first-generation methods. Current second-generation panel unit root methods, such as the cross-sectional Im, Pesaran and Shin (CIPS) unit root test and the cross-sectional augmented Dickey-Fuller (CADF) unit root test by Pesaran,^84^ can consider the cross-sectional dependence problem. The second generation of tests overturns N⁓∞ and performs well due to more advanced asymptotic premises. Thus, this study adopts the panel unit root method of CADF and CIPS to detect the dynamic integration level of the series to determine the robust results. The following equation is related to the CADF panel unit root process and is used to measure the level of integration of the panel dynamics.

(5) $$\Delta e_{\mathrm{it}}=\alpha_{i}+\beta_{i}{e_{i}}_{t-1}+\kappa_{i}{\overset{ˉ}{e}}_{t-1}+\psi \Delta {\overset{ˉ}{e}}_{t}+\mu_{\mathrm{it}}$$

By replacing the lag term (t-1) in Equation (5), the following equation can be established:

(6) $$\Delta e_{\mathrm{it}}=\alpha_{i}+\beta_{i}e_{\mathrm{it}}-1+\kappa_{i}{\overset{ˉ}{e}}_{t-1}+\sum_{j=0}^{k}\psi_{\mathrm{ij}}\Delta {\overset{ˉ}{e}}_{\mathrm{it}-1}+\sum_{j=0}^{k}\lambda_{\mathrm{ij}}\Delta e_{\mathrm{it}-1}+\upsilon_{\mathrm{it}}$$

In the above formula, $\Delta e_{\mathrm{it}-1}$ represents the hysteresis level of each section, and ${\overset{ˉ}{e}}_{\mathrm{it}-1}$ expresses the average value function related to the first-order derivative.

The following Equation (7) can be used to measure the dynamic integration level of the CIPS method.

(7) $$\mathrm{CIPS}=\;N^{-1}\;\sum_{i=1}^{N}\lambda_{i}\left(N,T\right)$$

$\lambda_{i}\left(N,T\right)$ in the above unit root measurement CIPS equation can be replaced by CADF to establish the new Equation (8).

(8) $$\mathrm{CIPS}=N^{-1}\sum_{i=1}^{N}\lambda_{i}\mathrm{CADF}$$

### 3.3. Westerlund^85^ Panel Long-Run Cointegration Procedure

An important step before estimating the long-run parameter estimates is to investigate the long-run cointegration relationships between the panel dynamics. Once the long-term relationship is identified, policymakers and officials can then carefully develop long-term strategies and regulations. Thus, this study adopts the second-generation cointegration test of Westerlund,^85^ which is more robust to data with cross-sectional dependence, structural breaks and small samples. Furthermore, one of the main benefits of using this strategy is that it can account for slope heterogeneity in panel data. The following is the econometric expression of the error correction model (ECM) cointegration test of Westerlund^85^

(9) $$\delta \;\mathrm{Zit}=\alpha_{i}d_{t}+\beta_{i}\left(X_{i,t-1}-\lambda_{i}Y_{i,t-1}\right)+\sum_{J=1}^{q}\beta_{\mathrm{ij}}\Delta Z_{i,t-j}+\sum_{J=0}^{q}\psi_{\mathrm{ij}}\Delta X_{i,t-j}+\varepsilon_{i,t}$$

In the above formula, $d_{t}$ = (1, t) ´ denotes intercept, $\alpha_{i}$_= (_$\alpha_{1,}$ $\alpha_{2}$) indicates the deterministic trend, i is the entire cross-section and t shows the time span.

The Group Westerlund technique described below can be used to measure long-term cointegration.

(10) $$G_{t}=\frac{1}{N}\sum_{i=1}^{N}\frac{\psi_{i}}{S.E.\left({\hat{\psi }}_{i}\right)}$$

(11) $$G_{a}=\frac{1}{N}\sum_{i=1}^{N}\frac{\psi_{i}}{\psi_{i}^{\prime }\left(1\right)}$$

The panel Westerlund process illustrated below can be used to measure long-term cointegration.

(12) $$P_{t}=\frac{{\hat{\psi }}_{i}}{S.E.\left({\hat{\psi }}_{i}\right)}$$

(13) $$P_{a}=T\;\;{\hat{\psi }}_{i}$$

Where, ${\hat{\psi }}_{i}$ mentioned in the Westerlund panel cointegration statistics above is the adjustment speed, which indicates the recovery of imbalance in the long term after the short-term discrepancy.

### 3.4. Cross Sectional Autoregressive Distributive Lag (CS-ARDL)

Consistent cross-sectional dependence of various determinants affecting all components can be found in panel data, such as various socioeconomic dynamics that may be affected by economic and financial shocks. Results obtained from regression analysis related to panel data with cross-sectional dependence and slope heterogeneity may be spurious or erroneous. Thus, to address the cross-sectional dependence and heterogeneous slope issues in panel data regression analysis, this study adopts the CS-ARDL procedure introduced by Chudik and Pesaran^86^ to reveal the short-term and long-term correlations between explanatory and explained dynamics in the proposed model. The core benefit of this procedure is to produce more robust results, regardless of whether the underlying parameters in the series are cointegrated, level stationery, first-difference stationery, or mixed integrals.^87,88^ Investigation by incorporating short-term and long-term effects can offer treasured perceptions into the progressive dynamics of economic and environmental interactions within newly industrialized countries. The mathematical form of the CS-ARDL method can be expressed as:

(14) $$y_{\mathrm{it}}=\;\sum_{i=0}^{pz}\theta_{l,i}y_{i,t-1}+\;\sum_{i=0}^{py}\beta_{l,i}V_{i,t-1}+\;\varepsilon_{\mathrm{it}}$$

Where $y_{\mathrm{it}}$ indicates explained factor, $y_{i,t-1}$, $\beta_{l,i}$, and $V_{i,t-1}$are the lag specification, explanatory dynamics parameter, and regressor dynamics lags at time *t*-1. In addition, $\varepsilon_{\mathrm{it}}$ is a random error term for time t and country i, describing unseen dynamics that may influence the explained factor. Furthermore, Equation (15) is modified by adding an additional term to take into account the average value of the explanatory factors across different cross-sections.

(15) $$y_{\mathrm{it}}=\sum_{i=0}^{pz}\theta_{l,i}y_{i,t-1}+\sum_{i=0}^{py}\beta_{l,i}V_{i,t-1}+\sum_{l=0}^{pw}w_{l,i}^{\prime }{\overset{ˉ}{x}}_{i,t-1}+\varepsilon_{\mathrm{it}}$$

Where ${\overset{ˉ}{x}}_{i,t-1}$ = ${\overset{ˉ}{y}}_{i,t-1}$ $V_{i,t-1}$ reflects the cross-sectional mean in lagged form, pz, py and pw represent the predictor dynamics in lagged form, and $\varepsilon_{\mathrm{it}}$ signifies the economy-specific error term taking into account separate effects in addition to the model-explained dynamics. Furthermore, the following Equations (16) and (17) represent the short-term and long-term model estimates obtained from the CS-ARDL method, respectively.

(16) $${\hat{\theta }}_{\mathrm{MG}}=\frac{1}{N}\sum_{i=1}^{N}{\hat{\theta }}_{i}\;\mathrm{and}\;\psi_{i}=\frac{\sum_{l=0}^{py}{\hat{y}}_{l,i}}{{\hat{\theta }}_{i}}$$

θ = −1$\left(1-\sum_{l=1}^{py}{\hat{y}}_{l,i}\right)$ denotes error correction mechanism (ECM), which measures model stability.

(17) $${\hat{\theta }}_{\mathrm{MG}}=\frac{1}{N}\overset{N}{\underset{i=1}{\sum }}{\hat{\theta }}_{i}\;\mathrm{and}\;{\hat{\theta }}_{\mathrm{CS}-\mathrm{ARDL},i}=\frac{\sum_{l=0}^{py}{\hat{y}}_{l,i}}{1-\sum_{l=1}^{pz}{\hat{\theta }}_{l,i}}$$

Where, estimation of each cross section can be denoted by θ as mentioned in the above equation. Equation (18) below demonstrates the CS-ARDL error correction mechanism.

(18) $$\Delta \mathrm{Zit}=\psi_{i}\left(z_{i,t-1}-{\hat{\theta }}_{i}y_{i,t-1}\right)-\alpha_{i}+\sum_{l=1}^{py-1}\beta_{l,i}\Delta_{l}Z_{i,t-l}+\sum_{l=0}^{px}\psi_{l,i}\Delta_{i}y_{i,t-1}+\sum_{l=0}^{pw}w_{l,i}{\overset{ˉ}{x}}_{i,t-1}+\varepsilon_{\mathrm{it}}$$

Where $\psi_{i}$ indicates the rate at which short-term imbalance is restored to long-term equilibrium through the error correction term (ECT).

### 3.5. Checking the Robustness of the CS-ARDL Approach

This study also uses the augmented mean group (AMG) approach to test the robustness of the long-term parameter estimation results of the CS-ARDL technique. The AMG process is similar to the CS-ARDL approach in that it fundamentally explores the dynamic links between transportation infrastructure, agricultural land use, agricultural value addition, environment-related technologies, and environmental contamination. The method addresses the intricacy of the envisioned multi-layered model and takes into account cross-sectional dependencies and potential variations over time. This process is integrated with rigorous econometric procedures to verify the robustness of inferences and to enhance the confidence and generalizability of the results. Robustness checks can support the interplay of insights from economic and environmental dynamics of densely populated economies in this study.

## 4. Analytical Results and Their Interpretation

The first step of the analysis in this study is to use diagnostic tests to detect problems in panel data, such as detecting cross-sectional correlation problems through cross-sectional dependency tests, perceiving heterogeneity problems through homogeneity tests, discovering the stationarity level of each panel dynamic through CIPS and CADF unit root methods, and finally revealing long-term cointegration through the method of Westerlund.^85^ The next step is to reveal the long-term parameter estimates by CS-ARDL, and finally the robustness of the CS-ARDL results can be checked by the AMG method. The estimation results in Table 2 illustrate that the overall dynamics of the model are significant through the cross-sectional dependence procedures of the Breusch-Pagan LM, the Baltagi-Feng-Kao bias-corrected scaling LM, Pesaran CSD, and the Pesaran scaled LM, so there is a problem of cross-sectional dependence in specific densely populated countries. The existence of cross-sectional correlation in panel data motivates us to use second-generation methods such as panel unit root, panel long-term cointegration and panel long-term parameter estimation to obtain more reliable, stable and robust results.^89,90^

**Table 2.** Results of the panel data cross-sectional correlation detection procedures.

Dynamics	Pesaran CSD		Pesaran Scaled LM		Bias-corrected Scaled LM		Breusch-Pagan LM	
	Statistics	P-value	Statistics	P-value	Statistics	P-value	Statistics	P-value
lnCO_2_	94.14**	0.053	53.08***	0.005	56.14***	0.007	842.04***	0.008
lnAGL	64.15***	0.007	62.57***	0.008	60.15***	0.006	966.77***	0.006
lnERT	93.68***	0.004	73.64***	0.005	64.66***	0.003	743.04***	0.009
lnAGV	65.10***	0.008	78.08***	0.006	50.03***	0.006	967.56***	0.009
lnGDP	42.65**	0.045	74.56**	0.055	73.58***	0.008	642.65***	0.008

*signifies a statistical significance level of 10%, **denotes a statistical significance level of 5%, and ***identifies a statistical significance level of 1%.

The dynamic descriptive statistics of the model in Table 3 show that the average carbon dioxide emissions of the top five countries in terms of population density are 16.18 billion tons, with a standard deviation of 5.48 billion tons during the period 1990–2022. Countries with higher emission levels tend to be those with higher population densities because these countries have higher demand for transportation, agricultural land use, and agricultural production activities, which lead to higher environmental emissions. The average GDP (market value of all final goods and services produced in a year) of the five countries with the highest population density is $239,689.47, and the average agricultural land use (% of land area) is 49.76%. The proportion of agricultural land in densely populated countries reflects that due to high population density, half of the land in these countries is used for agricultural purposes, resulting in higher environmental emissions. The average agricultural value-added accounts for 27.48% of GDP, and the average transportation infrastructure investment is US$36.54 billion. Furthermore, among the overall technologies, an average of 22.56% of environment-related technologies are being used by specific countries with higher population density. The share of environment-related technologies is quite low, making these countries vulnerable to higher environmental emissions.

**Table 3.** Descriptive statistics of model panel dynamics.

Dynamics	Average	SD	Max	Min	Kurtosis	Skewness	Jarqua-Bera	Probability
lnCO_2_	16.18	2.48	21.60	12.06	2.48	0.89	1.79	0.70
InAGL lnAGV	49.59 27.48	8.09 5.43	53.14 29.39	34.25 18.38	1.63 1.24	0.90 0.43	1.64 1.35	0.98 0.63
InTRI	36.54	4.38	32.19	28.19	1.94	0.61	1.53	0.82
InERT	22.56	2.46	25.59	8.37	1.57	0.99	1.86	0.94
InGDP	239689.47	11013.08	274874.53	147505.06	1.83	0.84	1.43	0.73

The correlation analysis of the model panel variables in Table 4 shows that agricultural land use, agricultural value added, transportation infrastructure, and GDP are gradually correlated with carbon dioxide emissions, while environment-related technologies are adversely correlated with environmental emissions. This correlation result evidently shows that the development of environment-related technologies can improve energy efficiency and thus alleviate environmental pollution by shifting from fossil fuels to clean energy. This study also employed the VIF test to unveil the multicollinearity problem of the model panel variables. The results show that the dynamic VIF statistics obtained are all lower than 5, and the tolerance values (TV) derived for each dynamic are all higher than 0.2, provide evidence that there is no multicollinearity problem in the panel dynamic model.

**Table 4.** Correlation and multicollinearity results in model panel dynamics.

	lnCO_2_	lnAGR	lnAGV	lnTRI	lnGDPlnERT	VIF	TV= (1/VIF)
lnCO_2_	1						
lnAGL lnAGV	0.763 0.598	1 0.547	1			3.50 2.87	0.285 0.348
lnTRI	0.867	0.709	0.358	1		2.07	0.483
lnGDP	0.609	−0.808	0.798	0.589	1	2.43	0.411
lnERT	−0.608	0.783	0.897	0.759	0.7981	2.97	0.336

In addition, using the homogeneity method of Pesaran and Yamagata^91^ to detect slope heterogeneity in panel dynamics data is also a crucial part of the analysis in this study, and the results are illustrated in Table 5. The results of the Pesaran and Yamagata heterogeneity test statistics in both models are significant, thus rejecting the null hypothesis of the existence of homogeneous slope coefficients in the model panel dynamics.

**Table 5.** Slope heterogeneity analysis using the pesaran and Yamagata procedure.

Models	Test	value	Probability
Model-CO_2_ (1)	Δ	18.81***	0.006
	${\tilde{\Delta }}_{\mathrm{adjusted}}$	17.73***	0.000
Model-CO_2_ (2)	Δ	19.74***	0.001
	${\tilde{\Delta }}_{\mathrm{adjusted}}$	15.39***	0.002

*signifies a statistical significance level of 10%, **denotes a statistical significance level of 5%, and ***identifies a statistical significance level of 1%.

The next step of the analysis is to uncover the unit root properties of each panel dynamics through the second-generation CIPS and CADF unit root methods. The results in Table 6 show that carbon dioxide emissions, agricultural value added and environment-related technologies do not have unit root problems, while agricultural land use, transportation infrastructure and gross domestic product (GDP) have unit root problems and are integrated at the first difference I(1). Evidence of mixed stationarity obtained through panel CIPS and CADF unit root methods allows for robust long-run parameter estimation using CS-ARDL.

**Table 6.** Using CADF and CIPS techniques to find panel unit roots.

Variables	CADF		CIPS	
	Level	First difference	Level	First Difference
lnCO_2_ lnAGL	−1.783*** −1.654	−1.674*** −3.439***	−2.013*** −1.591	−2.936*** −3.765***
lnAGV	−1.693***	−3.936***	−1.183***	−4.994***
lnTRI	−1.937	−2.094***	−1.974	−3.841***
lnGDP	−1.903	−3.839***	−1.876	−2.748***
lnERT	−1.793***	−2.967***	−1.278***	−2.837***

*signifies a statistical significance level of 10%, **denotes a statistical significance level of 5%, and ***identifies a statistical significance level of 1%.

The subsequent stage after establishing the unit root property of the panel dynamics is to reveal the long run cointegration relationship of the specific panel dynamics in the model. Hence, for this purpose, this study adopts the second generation Westerlund cointegration method. This procedure addresses the problems of cross-sectional dependence and slope heterogeneity in panel data and is the main reason for choosing this approach. The results highlighted in Table 7 show that the Westerlund group statistics Gt and Ga and the Westerlund panel statistics Pt and Pa are significant, confirming the long run cointegration of the panel dynamics in the series of densely populated countries.

**Table 7.** Results of estimating long-run cointegration using the Westerlund^85^ method.

Statistics	G_t_	G_a_	P_t_	P_a_
Values	−5.308***	−3.497***	−5.398***	−3.492
Z-values	−1.408	1.219	−2.409	1.113
P-values	0.001	0.148	0.002	0.191
Robust P-values	0.003	0.038	0.004	0.148

*signifies a statistical significance level of 10%, **denotes a statistical significance level of 5%, and ***identifies a statistical significance level of 1%.

Three inevitable conclusions can be drawn from the above basic diagnostic empirical analysis: First, environmental emissions in densely populated countries are strongly affected by the explanatory factors (agricultural land, agricultural value added, and transport infrastructure), as evidenced by the high correlations between the regressors and the dependent variable. Secondly, this study adopts the second-generation unit root methods of CIPS and CADF to reveal the stationarity level of each panel dynamics, as these procedures address the problems of slope coefficient heterogeneity and cross-sectional correlation in panel data. Finally, since the above diagnostic tests indicate that the panel data used for the analysis suffers from heterogeneity and cross-sectional dependence problems, these glitches can be addressed by using CS-ARDL and AMG as the most appropriate methods to estimate the long-run parameter estimates.

The long- term and short-term parameter estimation results for the CS-ARDL and AMG methods reported in Table 8 display that agricultural land use and investment in transportation infrastructure contribute significantly to CO_2_ emissions, but only in the long term. However, agriculture value addition strongly promotes environmental contamination in both the short and long term. The estimation results illustrate that each upsurge of 1 unit of agricultural land use, agricultural added value and transportation infrastructure investment can meaningfully induce carbon dioxide emissions in the long run, with increases of 0.398%, 0.336% and 0.246% respectively. The results evidently display that particular indicators such as agricultural land, agricultural value added, and transportation infrastructure in densely populated countries are severely relied on fossil fuel energy and are the core drivers of environmental emissions. The finding of the gradual influence of agricultural land on environmental contamination is align with those of Tacconi and Muttaqin^63^; Haider et al.^64^; Rehman et al.^65^; Gu, Li, and Santos^66^; Huang et al.^67^ Likewise, the results of the contribution of agricultural value added to environmental damage support the studies of Raihan^52^; Ali and Xiangyu^50^; Dimnwobi et al.^51^; Karimi Alavijeh et al.^53^; Usman et al.^54^; Lao and Luo.^56^ In addition, the result on the progressive influence of transportation infrastructure investment on environmental risks is consistent with those of Dai et al.^19^; Cao and Su^42^; Liu et al.^20^; Herath Bandara and Thilakarathne^43^; Wang et al.^44^; Pradhan et al.^45^ The estimation results show that densely populated countries have a robust inverted U-shaped EKC model based on the increasing effect of GDP on environmental pollution and the inverse effect of GDP square on environmental pollution but in the long run. More certainly, the adverse significance of the Error Correction Model (ECM) verifies the presence of long-run cointegration in the model panel dynamics. ECM measures the tendency of a variable to return to a state of equilibrium after a shock. The obtained ECM coefficient is −309, which means that 30% of short-term shocks can be recovered every year, indicating that ECM has strong resilience.

**Table 8.** Short-term and long-term parameter estimation results for the CS-ARDL and AMG procedures.

	CS-ARDL				AMG	
	Long term		Short term		Long term	
Panel dynamics	Parameters	Probability	Parameters	Probability	Parameters	Probability
CO_2_= f(AGL, AGV, TI, GDP, GDP^2^)						
lnAGL	0.398***	0.001	0.216	0.178	0.351***	0.005
lnAGV	0.336***	0.002	0.233*	0.059	0.274**	0.024
lnTI	0.246***	0.000	0.243	0.115	0.254**	0.019
lnGDP	0.284***	0.006	0.201	0.143	0.208***	0.007
lnGDP^2^	−0.154***	0.000	0.258	0.190	0.387***	0.002
C	0.497*	0.079	0.276	0.187	0.348**	0.038
R^2^	0.78		0.79	0.84		
ECM_(−1)_			−0.309***	0.000		
CO_2_= f(AGL, AGV, TI, GDP, ERT*AGL, ERT*AGV, ERT*TI)						
lnAGL	0.317***	0.000	0.229	0.107	0.369	0.003
lnAGV	0.259***	0.001	0.288*	0.062	0.247***	0.007
lnTI	0.275***	0.002	0.276	0.141	0.254***	0.005
lnGDP	0.396**	0.019	0.270	0.119	0.162	0.189
lnERT*AGL	−0.298***	0.000	−0.128	−0.154	−0.328***	0.000
lnERT*AGV	−0.315***	−0.001	−0.214**	−0.027	−0.214***	−0.005
lnERT*TI	−0.285***	−0.005	−0.169	−0.319***	−0.007	−0.169
C	0.315***	0.001	0.217***	0.008	0.239***	0.000
R^2^	0.68		0.73	0.79		
ECM_(−1)_			−0.247***	0.001		

*signifies a statistical significance level of 10%, **denotes a statistical significance level of 5%, and ***identifies a statistical significance level of 1%.

Environmentally related technologies improve energy efficiency and enable a shift from traditional fossil fuel transportation to cleaner, more sustainable alternative fuels, thereby improving environmental quality and promoting a sustainable future. Likewise, environment-related technologies improve energy efficiency by providing renewable energy sources such as biofuels, wind and solar energy for use in the agricultural sector, thereby mitigating greenhouse gas emissions. Thus, environment-related technologies indirectly affect environmental pollution through interactions with agricultural land use, agricultural value addition, and transportation infrastructure. The estimated results of the moderating role of environment-related technologies in the association between explanatory and explained dynamics regression indicate that the interaction of environment-related technologies with agricultural land use and transport infrastructure can significantly mitigate CO_2_ emissions, but only in the long run. However, the interaction of environment-related technologies and agricultural value addition can deeply degrade ecological contamination in the short and long term. It is evident from the analysis that agricultural land use, agricultural value addition, and transportation infrastructure without interaction with environment-related technologies can aggravate environmental hazards, whereas these dynamics interacting with environment-related technologies can mitigate environmental risks. Hence, this study proposes that densely populated countries can achieve environmental sustainability goals by developing environmentally relevant technologies that can generate clean fossil fuel-free energy for use in the agriculture and transportation sectors.

In addition, this study estimates the threshold extent of environmentally relevant technologies that have nonlinear effects on ecological hazards through interactions with agricultural land use, agricultural value addition, and transportation infrastructure. Estimated threshold regression ranges for environmentally relevant technologies allow the selection of specific threshold levels, which may have a strong and nonlinear effect on environmental pollution. The estimated threshold regression results in Table 9 show that −1.115 is the threshold level of environment-related technologies. The estimated environmental-related technology thresholds are more effective for the impacts of agricultural land use, agricultural value added, and transportation infrastructure on environmental damage. When the threshold value of environment-related technology is low (≤-1.115), the effects of agricultural land use, agricultural value added and transportation infrastructure on ecological damage are not robust, but when the threshold value is high (>-1.115), the effects of these important regressors on environmental damage become significant.

**Table 9.** Threshold regression estimation results.

Environment related technologies		
	Threshold one	Threshold two
	≤ − 1.115	> − 1.115
lnAGL	−0.038 (0.183)	−0.069*** (0.005)
lnAGV	−0.052 (0.115)	−0.085*** (0.008)
lnTRI	−0.069 (0.147)	−0.061*** (0.003)
Threshold regression estimation		
RSS	18.639	
F. statistics	81.86 [0.002]	

*signifies a statistical significance level of 10%, **denotes a statistical significance level of 5%, and ***identifies a statistical significance level of 1%. The values in brackets are the probabilities of each statistic.

In summary, in the top five economies with higher population density, environment-related technologies can indirectly and effectively reduce environmental pollution through interactions with agricultural land, agricultural value added, and transportation infrastructure. Developing environment-related technologies can improve energy efficiency by providing renewable energy sources such as biofuels, wind and solar energy to the agricultural and transportation sectors, thereby alleviating environmental pollution.

### 4.1. Review to Check Robustness

A second-generation AMG process is also employed to test the robustness of the long-term parameters estimated by the CS-ARDL method. The results of the AMG approach in Table 8 display that agricultural land use and transport infrastructure investment contribute meaningfully to CO_2_ emissions, but only in the long term, while agricultural value added strongly contributes to environmental damage in both the short and long term. The link between economic growth and environmental harm suggests an inverted U-shaped EKC hypothesis for specific densely populated countries, but only in the long run. Environmentally relevant technologies can indirectly and heterogeneously mitigate CO_2_ emissions through interactions with agricultural land use, agricultural value-added, and transport infrastructure investment. The results of estimating long-term parameters by the AMG technique are very consistent with those obtained by the CS-ARDL process.

## 5. Conclusion and Policy Recommendations

Densely populated countries have greater demand for agricultural products, greater vehicle usage, and busier traffic, so these countries need to use more agricultural land for agricultural production and invest more in transportation infrastructure, leading to higher environmental emissions. The study suggests that using environmentally friendly technologies based on renewable energy in the transportation and agricultural sectors can cut down environmental emissions. Thus, this study determines to disclose the influence of agricultural land use, agricultural value added, and transportation infrastructure investment on ecological damage in countries with the highest population density; and the sole moderating effect of environment-related technologies in the association between agricultural land use, agricultural value added, transportation infrastructure investment and CO_2_ emission. In addition, this study also estimates the threshold extent of environmentally relevant technologies that have nonlinear effects on ecological hazards through interactions with agricultural land use, agricultural value addition, and investment in transportation infrastructure. Hence, in terms of estimation, this study adopts the CS-ARDL process to estimate short-term and long-term parameters, and uses the AMG approach to test the robustness of the CS-ARDL long-term results.

The long- term and short-term parameter estimation results for the CS-ARDL and AMG methods display that agricultural land use and investment in transportation infrastructure contribute significantly to CO_2_ emissions, but only in the long term. However, agriculture value addition strongly promotes environmental contamination in both the short and long term. The estimation results also show that densely populated countries have a robust inverted U-shaped EKC assumption based on the increasing effect of GDP on environmental contamination and the inverse effect of GDP square on environmental effluence but in the long run. The moderating role of environment-related technologies in the association between explanatory and explained dynamics regression indicate that the interaction of environment-related technologies with agricultural land use and transport infrastructure can significantly mitigate CO_2_ emissions, but only in the long run. However, the interaction of environment-related technologies and agricultural value addition can deeply degrade ecological contamination in the short and long term. It is evident from the analysis that agricultural land use, agricultural value addition, and transportation infrastructure without interaction with environment-related technologies can aggravate environmental hazards, whereas these dynamics interacting with environment-related technologies can mitigate environmental risks. The estimated environmental-related technology thresholds are more effective for the impacts of agricultural land use, agricultural value added, and transportation infrastructure on environmental damage. When the threshold value of environment-related technology is low, the effects of agricultural land use, agricultural value added and transportation infrastructure on ecological damage are not robust, but when the threshold value is high, the effects of these important regressors on environmental damage become significant.

Environmental sustainability goals can be effectively accomplished by progressing strong policies based on the empirical findings of the current study. First, densely populated countries can adopt environment-related technologies to improve energy efficiency by producing renewable energy, thereby reducing environmental emissions. By developing environmentally relevant technologies to install more renewable energy sources to cope with the depletion of existing fossil fuels, a sustainable development environment can be established in densely populated countries. Higher economic growth relies on higher levels of fossil fuels as the main production input, but on the other hand, fossil fuel energy pollutes the environment, reflecting the reality that sustainable growth comes at the expense of a high-quality environment. Thus, the goals of sustainable environment and economic growth can be achieved by focusing more on renewable energy as an auspicious alternative to fossil fuels, which can be generated by developing environment-related technologies. Second, the empirical findings of the study evidently exhibit that unsustainable transportation and conventional agricultural practices in densely populated countries are severe obstacles to environmental sustainability. Basically, densely populated countries should prioritize the adoption of environmentally friendly technologies in agriculture and transportation to achieve environmental sustainability. Policymakers should prioritize using environmentally friendly building materials, adding new facilities or renovating old roads rather than traditional investments in polluting road and aviation infrastructure. Furthermore, investment in green research can promote the development of environmentally friendly technologies based on clean energy for use in agriculture and transportation, ensuring lower emission levels and a sustainable environment.

From the empirical research, this study proves that agricultural land use, agricultural value added and investment in transportation infrastructure are the main drivers of environmental pollution. However, there are other important components of agricultural and transport infrastructure, such as livestock production, rice production, inorganic fertilizer use, road infrastructure, aviation infrastructure, railway infrastructure, and inland waterway infrastructure that should be tested in future studies as major drivers of environmental emissions. Strategically, the moderating role of environment-related technologies should also be indirectly tested in the impact of disaggregated indicators such as livestock production, rice production, inorganic fertilizer use, road infrastructure, aviation infrastructure, railway infrastructure, and inland waterway infrastructure on environmental pollution in densely populated countries.

## Data Availability

The panel data used in the current study for analysis can be retrieved through at https://databank.worldbank.org/reports.aspx?source=World-Development-Indicators. Renewable energy consumption data can be retrieved through https://data.oecd.org/energy/renewable-energy.htm., while transport infrastructure investment data can be found at https://data.oecd.org/transport/infrastructure-investment.htm.
